# New insight into a simple high-yielding method for the production of fully folded and functional recombinant human CCL5

**DOI:** 10.1038/s41598-024-75327-y

**Published:** 2024-10-15

**Authors:** Afzaal Tufail, Saeed Akkad, Amanda R. Noble, Martin A. Fascione, Nathalie Signoret

**Affiliations:** 1 Hull York Medical School, University of York, York, YO10 5DD UK; 2 Department of Chemistry, University of York, York, YO10 5DD UK

**Keywords:** CCL5, CCR5, Recombinant chemokine production, Protein purification, Chemokine activity testing, Biological techniques, Cell biology, Immunology

## Abstract

Chemokines are proteins important for a range of biological processes from cell-directed migration (chemotaxis) to cell activation and differentiation. Chemokine C-C ligand 5 (CCL5) is an important pro-inflammatory chemokine attracting immune cells towards inflammatory sites through interaction with its receptors CCR1/3/5. Recombinant production of large quantities of CCL5 in *Escherichia coli* is challenging due to formation of inclusion bodies which necessitates refolding, often leading to low recovery of biologically active protein. To combat this, we have developed a method for CCL5 production that utilises the purification of SUMO tagged CCL5 from *E. coli* SHuffle cells avoiding the need to reform disulfide bonds through inclusion body purification and yields high quantities of CCL5 (~ 25 mg/L). We demonstrated that the CCL5 produced was fully functional by assessing well-established cellular changes triggered by CCL5 binding to CCR5, including receptor phosphorylation and internalisation, intracellular signalling leading to calcium flux, as well as cell migration. Overall, we demonstrate that the use of solubility tags, SHuffle cells and low pH dialysis constitutes an approach that increases purification yields of active CCL5 with low endotoxin contamination for biological studies.

**Supplementary Information:**

The online version contains supplementary material available at 10.1038/s41598-024-75327-y.

## Introduction

**Fig. 1:**
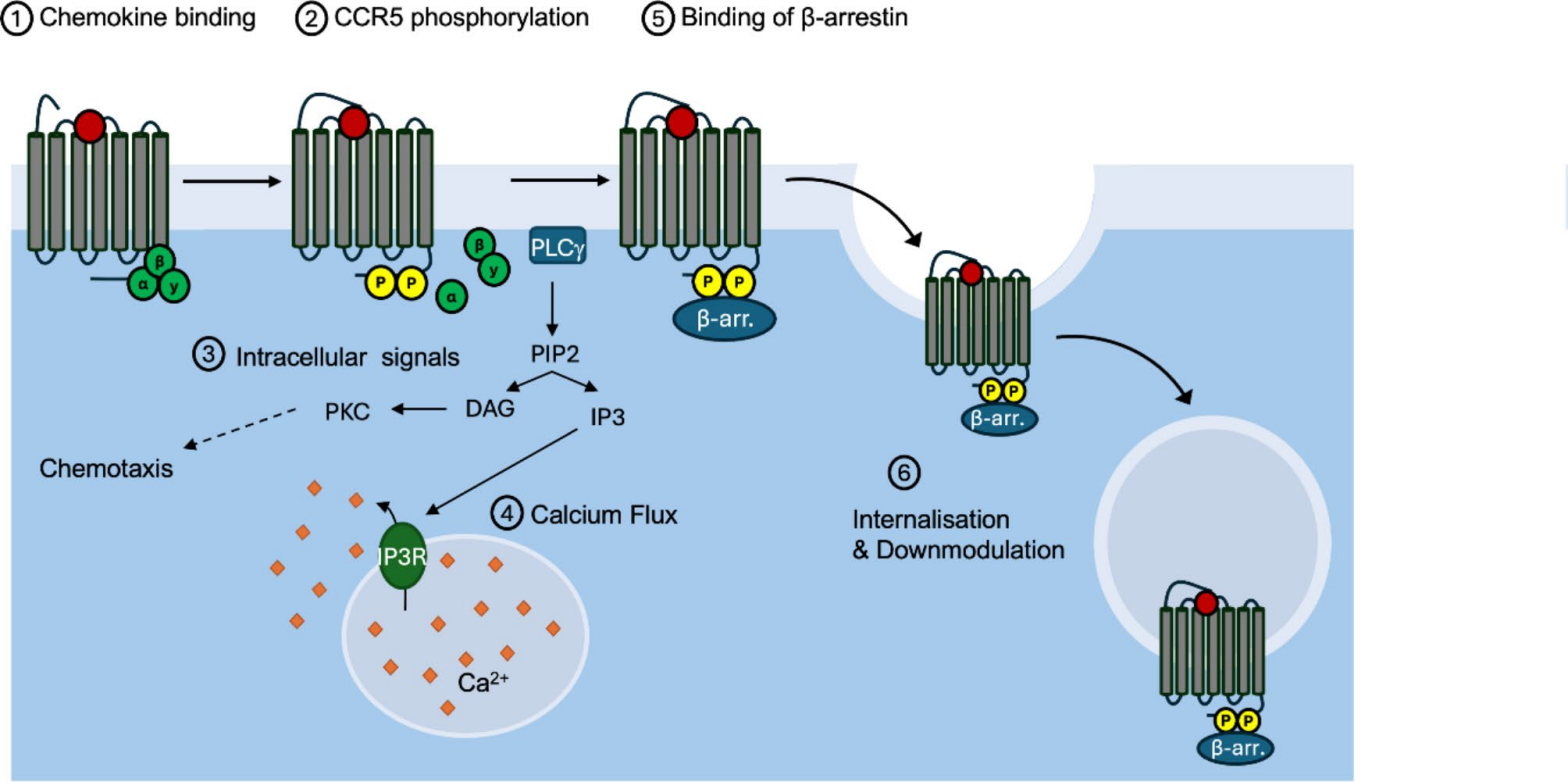
CCL5-mediated activation of CCR5. Ligand binding to CCR5 induces receptor phosphorylation, intracellular signals and calcium flux before removal of ß-arrestin (ß-arr.)-bound surface receptors by internalisation leading to CCR5 downmodulation.

Chemokines, or chemotactic cytokines, are small proteins (8–14 kDa) categorised into four subclasses (C, CC, CXC CX_3_C) based on the arrangement of conserved cysteine residues forming disulfide bonds, which maintain the tertiary structure required for their functional activity^1^. Chemokines can mediate a wide range of biological functions from cell-directed migration (chemotaxis) to cell activation and differentiation by triggering intracellular signalling upon interaction with cell-surface G-protein coupled receptors (GPCRs) known as C, CC, CXC and CX_3_C chemokine receptors. The CC chemokine CCL5, also known as RANTES (regulated on activation, normal T cell expressed and secreted) is a proinflammatory agonist of the CC chemokine receptors 1, 3 and 5 (CCR1, CCR3, and CCR5) responsible for the recruitment of lymphocytes, monocytes as well as eosinophils to sites of inflammation^2^. CCL5 interacts with CCR5 in a two-step process initially via ionic interactions between the chemokine core region and negative charged residues on the extracellular surface of CCR5^1^. This induces conformational changes in the CCR5 allowing activation of intracellular αβγ heterotrimeric G-proteins, with α and β/γ subunits separately initiating downstream signals engaging second messengers and a variety of kinases (see Fig. 1) and triggering calcium flux^1^. Meanwhile, the cytoplasmic tails of CCL5-bound receptors are phosphorylated by a G-protein Receptor Kinase (GRK), before β-arrestin recruitment and clathrin-mediated endocytosis of activated receptors for desensitisation^3^. The recruitment of β-arrestin to CCR5 halts G-protein signals and triggers additional β-arrestin-dependent signalling^1^. Overall signalling induced by CCL5-dependent activation of CCR5 can lead to a wide range of functional effects, from cytoskeletal rearrangements needed for migration, to changes in gene expression during cell activation, and/or regulation of metabolism leading to cell proliferation^4–7^. The CCL5/CCR5 axis plays a vital role in host responses to infection and inflammation, and has also been implicated in numerous pathological conditions including atherosclerosis, inflammatory bowel disease, as well as cancer where it is proposed to impact on tumour progression and metastasis^8,9^. However, our understanding of the molecular mechanisms underpinning CCL5/CCR5 activities in pathological conditions is still limited and demands further investigations before considering effective therapeutic interventions^10,11^.

To carry out studies of CCL5-dependent CCR5 activities in cells, scientists largely rely on the supply of bioactive recombinant human CCL5 produced in bacteria. To date, the main strategies for production use BL21 *E. coli* and are plagued by low purification yields due to CCL5 gene expression resulting in mainly misfolded insoluble chemokines within inclusion bodies. CCL5 expression in *E. coli* results in irregular formation of the structural disulfide bonds engaging the first two cysteines (C_10_ and C_11_), in the centre (30s loop) and the C-terminus (50s loop) of the protein, respectively^12^. Studies have overcome the issue of misfolded chemokines by recovery form inclusion bodies through a process of solubilisation in the presence of guanidine hydrochloride followed by refolding^13^. However, these steps are labour and resource intensive, increase purification timelines and can yield poor recovery of bioactive chemokines, including CCL5^14^. One way to overcome solubility issues is by using tags such as Maltose binding protein (MBP), Glutathione S-transferase (GST) or Small ubiquitin-like modifier (SUMO), which can increase recombinant protein solubility^15^ and have previously been exploited for the production of recombinant CCL5^14,16,17^. The MBP tag has been shown to be more efficient than GST for CCL5 when incorporated at the *N*-terminus^16^, with evidence that *C*-terminal tags can affect CCL5 function^14^. The in vitro refolding process can be avoided using SHuffle lysY T7 *E. coli*, a strain engineered to constitutively express disulfide isomerase DsbC, which can assist with correct folding of proteins forming multiple disulfide bonds in the cytoplasm^18^. The use of an *N*-terminal His-SUMO tag and expression in SHuffle cells has been reported to facilitate recombinant production of chemotactically active CXCL8 and CXCL17 as a mix of monomeric and dimeric forms^19,20^. For CCL5, evidence indicates that monomers activate CCR5^21–23^, and a major issue with production of recombinant CCL5 in *E. coli* is its propensity to aggregate at high concentration leading to precipitation of the protein^24^.

In the present study, we tailor the production strategy combining SHuffle *E. coli* expression and a N-terminal His-SUMO tag fusion protein, to achieve rapid high-yield of properly folded full-length human CCL5. The biological activity of the purified ‘in-house’ CCL5 (IH-CCL5) was assessed using established assays reporting on individual mechanistic steps underpinning CCL5-mediated activation of CCR5 in CHO-CCR5 cells^25–28^. These assays showed a comparable functional activity of IH-CCL5 and commercially sourced recombinant CCL5. Overall, we report on a simple and effective method to produce high quantities (milligrams) of pure and fully functional human CCL5 *‘in-house’* without refolding.

## Results

### Production of high yield recombinant IH-CCL5

**Fig. 2:**
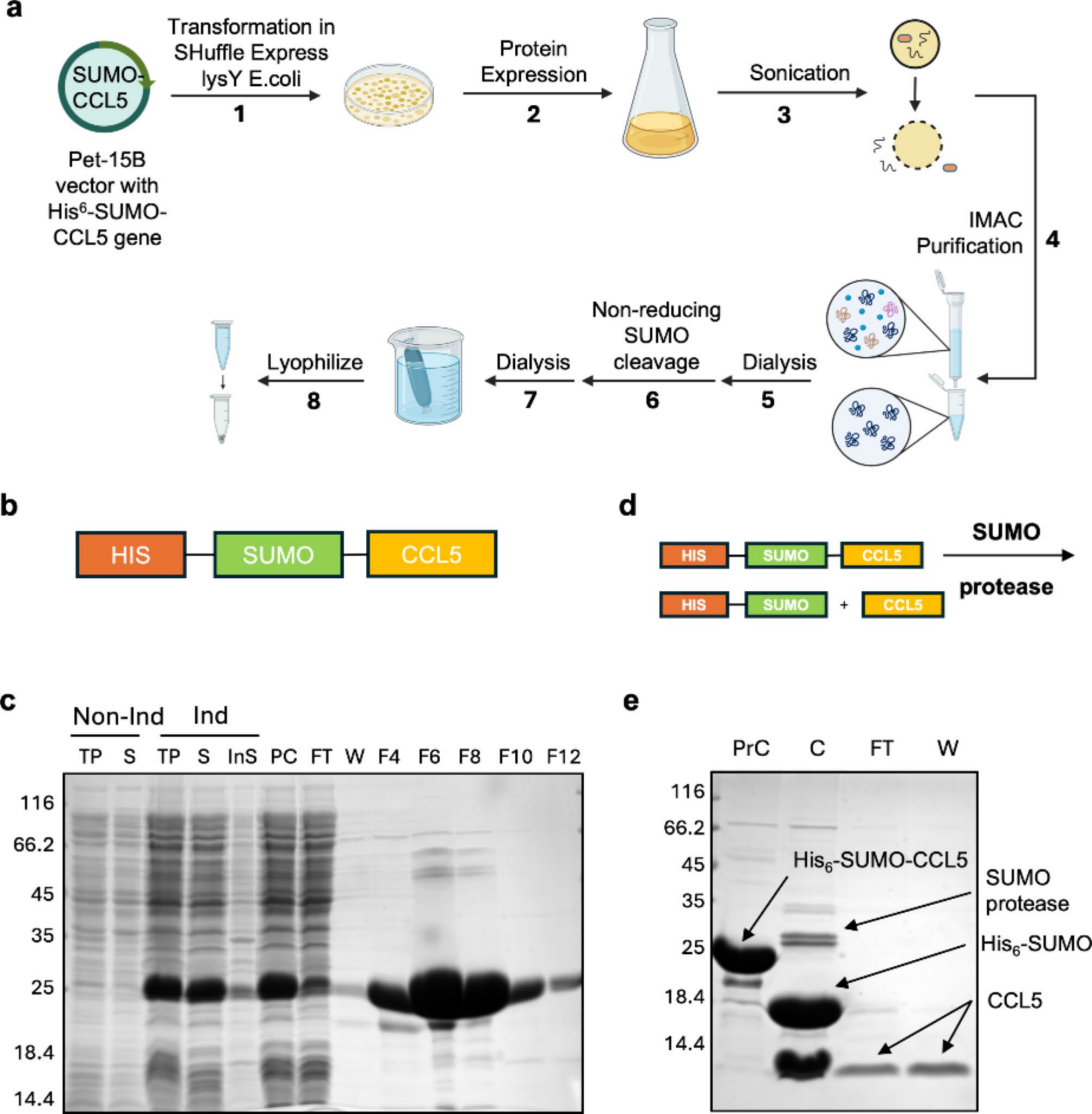
Purification of recombinant IH-CCL5 using Shuffle lysY cells. (**a**) Workflow of IH-CCL5 purification, figure generated using BioRender. (**b**) His_6_-SUMO-CCL5 construct representation. (**c**) SDS-PAGE analysis of SUMO-CCL5 post Ni^2+^ purification. Lane representation follows lane 1: non-induced total protein (TP), lane 2: non-induced soluble (S), lane 3: induced TP, lane 4: induced S, lane 5: induced insoluble (Ins), lane 6: pre-column, lane 7: His-column flow though, lane 8: His-column wash, lane 9–13: elution fractions F4,6,8,10 and 12. (**d**) SUMO cleavage of His_6_-SUMO-CCL5 construct representation. (**e**) SDS-PAGE analysis post nickel column purification of SUMO cleaved CCL5. Lane 1: His_6_-SUMO-CCL5 pre-cleavage (PrC), lane 2: cleaved CCL5 (C), lane 3: column flow-through (FT) and lane 4: column wash (W).

The His-SUMO-CCL5 fusion protein was overexpressed by plasmid (supplementary Fig. 1) transformation into SHuffle lysY T7 *E. coli* and recovered following a four-hour IPTG induction (Fig. 2a), as previously described^20^. Successful purification of the His-tagged protein was confirmed post Ni^2+^ affinity chromatography using SDS-PAGE, yielding approximately 120 mg of His-SUMO-CCL5 per litre of bacterial culture (Fig. 2b-c). The His-SUMO solubility tag was then cleaved using a ubiquitin-like-specific protease 1 (ULP1) in a non-reducing environment, in order to maintain the ternary structure of the CCL5 (Fig. 2d). SUMO proteases can form reversible intermolecular disulfide bonds^29,30^. Reducing agents such as dithiothreitol (DTT) are therefore used to maintain a reduced environment, increasing the activity of the protease and reducing cleavage time. However, CCL5 contains structural disulfides and therefore the addition of reducing agents causes the protein to misfold. SDS-PAGE analysis confirmed the successful DTT-free cleavage and separation of IH-CCL5 ( ~ 8 kDa) from the cleaved mixture (Fig. 2e). Successful production of IH-CCL5 was confirmed using liquid chromatography coupled with mass spectrometry (LC-MS). An experimental protein mass of 7847.47 Da (expected mass 7851.01 Da) was observed, revealing the purification of CCL5 with both disulfide bonds formed (supplementary Fig. 2). The purification yielded (~ 25 mg/L) of IH-CCL5, which was dialysed in successive steps into lower pH conditions. This included dialysis into 50 mM NaOAc (pH 4.5) with 500 mM NaCl overnight, then into 1% acetic acid (AcOH) overnight, finally into 0.1% trifluoroacetic acid (TFA) overnight and then lyophilised and kept at -80^o^C for long term storage. This slow acidification process favours the existence of CCL5 as monomers/dimers known to dominate below pH 4.0, while the oligomeric state is favoured at pH 4.0–5.0, and a pH above 5.0 can lead to CCL5 precipitation^31^. Endotoxin testing of purified IH-CCL5 by LAL assay (see method), reported a low level of contamination similar to commercially prepared recombinant CCL5 (≤ 1EU/ug of protein).

To assess the biological activity of produced IH-CCL5, we sought to test the activity of the chemokine using a number of biological assays reporting on mechanistic steps associated with CCR5 activation (Fig. 1). We compared the effects of IH-CCL5 and commercially sourced human CCL5 on a Chinese Hamster Ovary (CHO) cell line stably transfected to express functional CCR5 (CHO-CCR5)^25–28^.

### IH-CCL5 binding to cell-surface CCR5 and induction of CCR5 phosphorylation

**Fig. 3:**
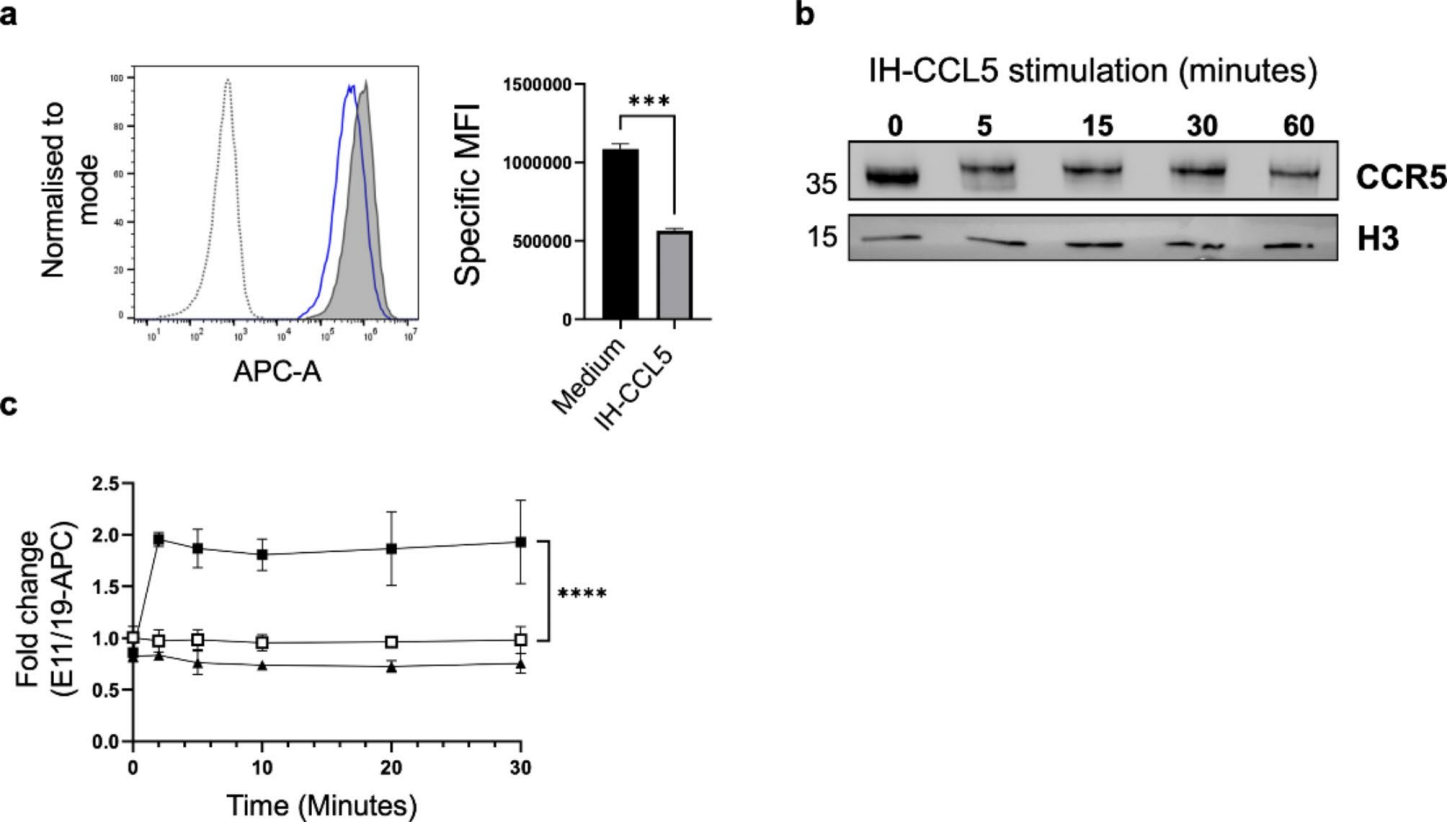
IH-CCL5 binding induces CCR5 phosphorylation on CHO-CCR5 cells. IH-CCL5 binding and kinetics of CCR5 phosphorylation using IH-CCL5 over the course of 30 min. (**a**) Flow cytometry analysis of IH-CCL5 binding to CCR5, assessed by loss of anti-CCR5 2D7 signal. Histograms for cells in medium (grey filled) and 100 nM IH-CCL5 (blue solid line) overlayed with no 2D7 (grey dotted line). Bar chart displaying change in specific MFI between medium and IH-CCL5 treated cells. Data shown from a representative experiment. Data analysed with t-test *** *P* < 0.0002. (**b**) CHO-CCR5 immunoblot after 100 nM IH-CCL5 stimulation for up to 60 min. Anti-CCR5 mAb MC5 (1 µg/mL) was used to detect CCR5 and histone-3 (H3) as a loading control (full-length blot in supplementary Fig. 4). (**c**) Fold change in E11/19-APC signal (phospho-FLOW) over 30 min CCR5 stimulation with 100 nM IH-CCL5 (*n* = 3). **** *P* ≤ 0.0001 two-way ANOVA. Graph symbols medium (open square), IH-CCL5 (filled square) and IH-CCL5/MVC (filled triangle).

CCL5 binding to CCR5 is characterised by an interaction involving the *N*-terminus of the receptor as well as its second extracellular loop (ECL2) housing the main chemokine binding site. ECL2 is also the binding site for an anti-CCR5 monoclonal antibody (2D7), shown to compete with CCL5 for CCR5 binding^32–34^. We carried out a similar competition assay^32,34^ to assess the binding of IH-CCL5 on CHO-CCR5 cells by flow cytometry. Cells treatment with 100 nM IH-CCL5 showed a significant reduction in 2D7 cell-associated fluorescent signal (Fig. 3a) with a 51.9% +/- 1.4% loss of antibody binding compared to non-treated cells, indicative of CCR5 occupancy by IH-CCL5.

IH-CCL5-induced CCR5 activation was observed indirectly through immunoblot analysis (Fig. 3b and supplementary Fig. 3), whereby stimulation of CHO-CCR5 cells leads to a band shift in CCR5 molecular weight associated with ligand stimulation linked to phosphorylation events^27,35^. The cytoplasmic tail of CCR5 contains serine residues, which are phosphorylated in response to chemokine stimulation (Fig. 1). G-protein receptor kinases (GRK) are known to specifically target the Ser^349^ residue following CCL5-mediated activation^36^. This phosphorylation can be detected with an anti-CCR5 monoclonal antibody (E11/19), which specifically recognises the phosphorylated Ser^349^ residue^36^. Briefly, we performed a kinetic analysis of CHO-CCR5 cells treated with IH-CCL5 using a Phospho-Flow cytometry approach^35^ measuring E11/19 staining overtime on fixed and permeabilised cells. An increase in E11/19 staining was detected with IH-CCL5 after 2 min of stimulation and was sustained over the 30 min, and was abrogated for cells pre-incubated with the CCR5 specific antagonist maraviroc (MVC)^37^, confirming that the phosphorylation is a result of IH-CCL5-induced activation of CCR5 (Fig. 3c and supplementary Fig. 4).

### IH-CCL5 induces downstream signalling that leads to calcium release and cell migration

**Fig. 4:**
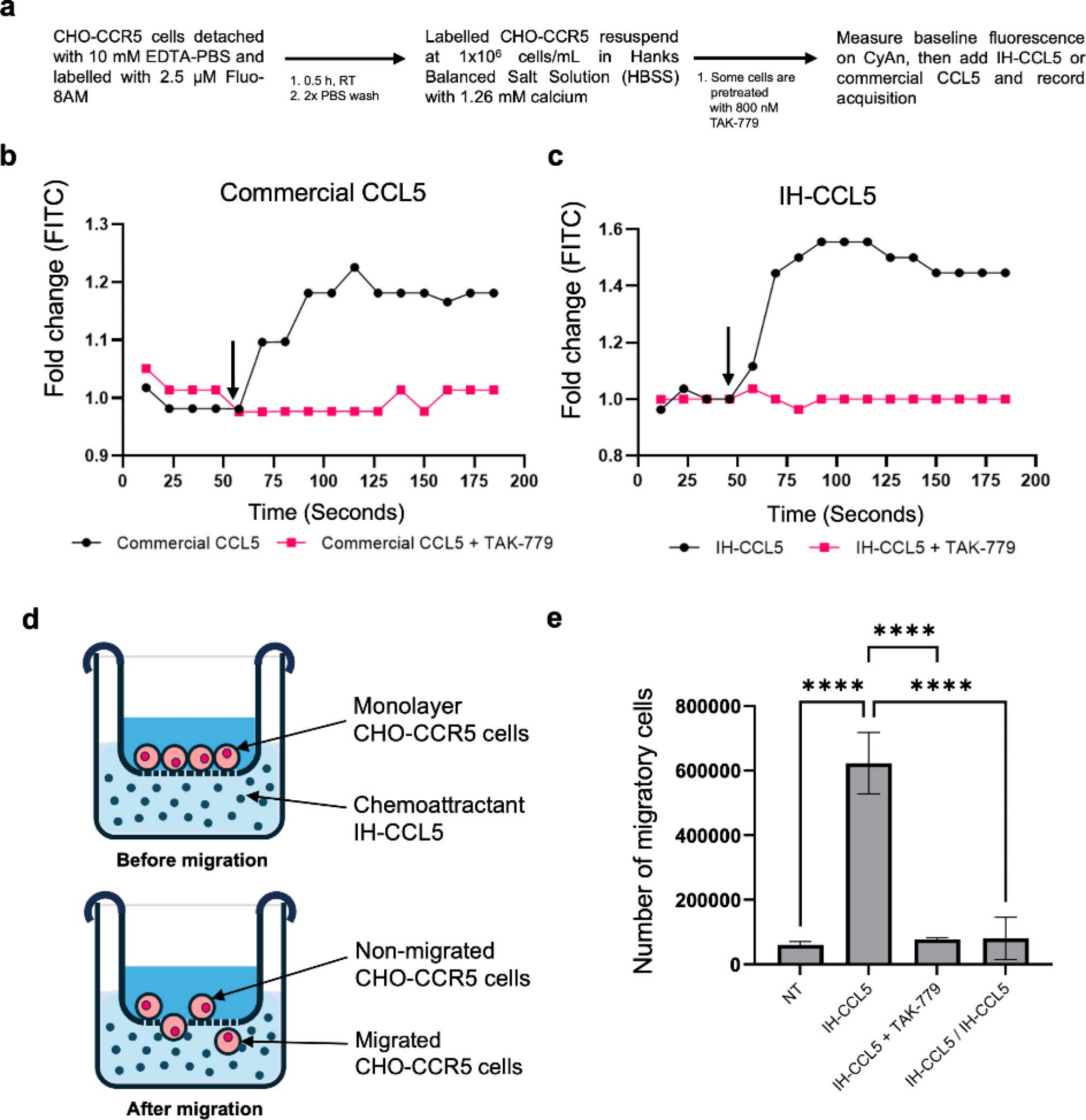
In-house CCL5 induced downstream CCR5 signalling activity. Chemokine binding activates downstream signalling that facilitates the release of calcium and cell migration. (**a**) Flow cytometry based experimental workflow for measuring calcium release upon chemokine stimulation. Cells were loaded with Fluo-8 AM (calcium reporter dye) and stimulated with 100 nM chemokine +/- TAK-779 and calcium release was measured using CyAn flow cytometer (FITC). Arrows on the graphs show time at which (**b**) 100 nM commercial CCL5 or (**c**) 100 nM IH-CCL5 were added. Results are from a representative experiment. CCL5 induced activation of CCR5 induces downstream signalling, which is involved in cell migration. (**d**) IH-CCL5 mediated CHO-CCR5 migration was assed using a Transwell migration assay with a 12 μm polycarbonate membrane pore size. (**e**) CHO-CCR5 cells were stimulated with 10 nM IH-CCL5 with or without 800 nM TAK-779. Cell migration was determined as the number of cells that migrated through the membrane filter into lower chamber (*n* = 3). One-way ANOVA statistical analysis with Bonferroni test was performed on the data **** *P* < 0.0001.

CCR5 activation process involves the stimulation of intracellular pathways through phosphorylation of downstream kinases and the release of calcium from intracellular store^38,39^. We investigated IH-CCL5 ability to trigger calcium flux using live flow cytometry analysis of CHO-CCR5 cells loaded with the cell permeable Fluo-8AM calcium reporter dye, measuring increase in fluorescence signal overtime due to intracellular calcium binding, as previously reported (Fig. 4a)^35^. Release of calcium was observed upon cell simulation with 100 nM of commercial CCL5 (Fig. 4b) as well as 100 nM of IH-CCL5 (Fig. 4c) in these assays, thus confirming that IH-CCL5 is as effective as commercial CCL5. The calcium signal was abrogated in the presence of the CCR5 antagonist TAK-779 (800 nM), confirming that the IH-CCL5-induced release was CCR5-dependent (Fig. 4b and c). Note that IH-CCL5-mediated calcium was detectable upon treatment with 10 nM as well as 100 nM IH-CCL5 (supplementary Fig. 5).

Ligand-mediated CCR5 activation and downstream signalling lead to a cell migratory response when subject to a chemotactic gradient^35^. The ability of IH-CCL5 to induce migration of CHO-CCR5 cells was tested using a Transwell migration assay where the cells would migrate through a polycarbonate membrane (Fig. 4d). A 10 nM IH-CCL5 simulation of CHO-CCR5 cells led to a sharp increase (~ 10-fold) in migration compared to non-stimulated CHO-CCR5 cells (Fig. 4e). Chemotaxis was abrogated by cell pre-treatment with TAK-779, or when IH-CCL5 was added at 10 nM in both upper and lower compartments neutralising the chemokine gradient (Fig. 4e). Overall, we show that IH-CCL5-stimulation supports chemotaxis.

### IH-CCL5 stimulation triggers downmodulation of cell surface CCR5 via endocytosis

**Fig. 5:**
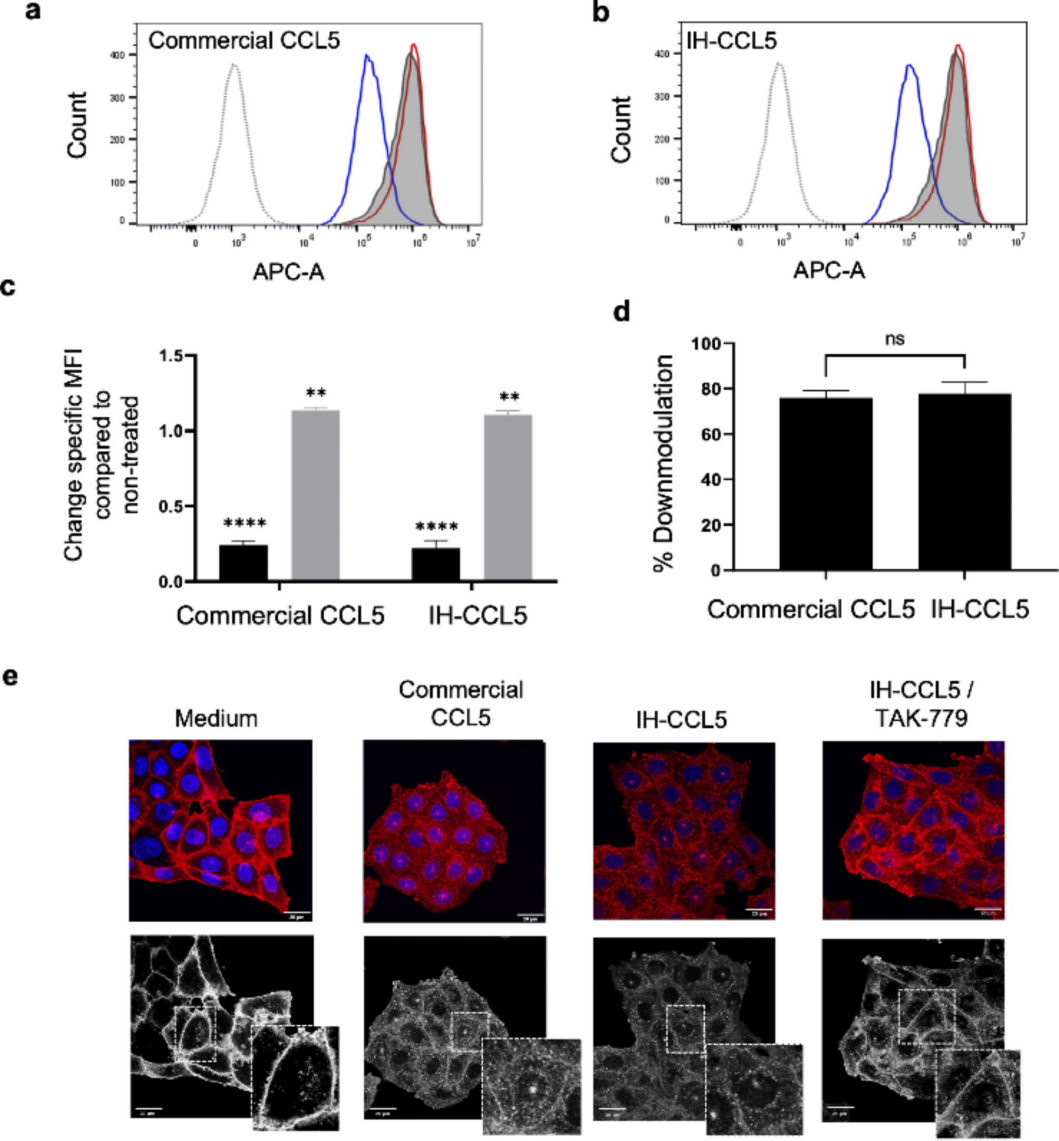
IH-CCL5 induced CCR5 downmodulation and internalisation. CCR5 downmodulation was assessed through the loss of anti-CCR5 MC5 binding at 37 ^o^C. (**a**,**b**) Flow cytometry histogram overlays for MC5 (detected with an anti-mouse A647; APC channel) in different conditions (Medium – Solid grey line with grey fill, 100 nM CCL5 – Blue solid line, 100 nM CCL5 + 800 nM TAK-779 – Red solid line and isotype control – Grey dotted line). (**c**) Change in specific MFI following treatment with the indicated recombinant CCL5 alone (black bars) or with TAK-779 pre-treatment (Grey bars) compared to untreated cells (*n* = 3). ** *P* ≤ 0.0021 and **** *P* ≤ 0.0001 One-way ANOVA with Bonferroni test. (**d**) Comparison of CCR5 downmodulation induced by IH-CCL5 and commercial CCL5 for CHO-CCR5 cells treated with 100 nM chemokine 1 h at 37^o^C (*n* = 3). T-test showed no significant difference (ns). (**e**) IH-CCL5 induced CCR5 internalisation using immunofluorescence microscopy. CHO-CCR5 cells were prelabelled with MC5 (5 µg/mL) followed by 100 nM chemokine treatment 0.5 h 37^o^C. Secondary anti-mouse A568 4 µg/mL (red) and cells mounted in mowiol containing DAPI (blue). Confocal microscopy images (scale bar at 20 μm) analysed on ImageJ.

Downmodulation is a “protective” process whereby activated GPCRs are removed from the cell surface to prevent prolonged stimulation and downstream receptor associated signalling, known as GPCR desensitization^40^. Post CCR5 activation and phosphorylation, β-arrestin proteins bind to the phosphorylated cytoplasmic tail (CCR5) and trigger removal from the cell surface^3^. CCR5 downmodulation results from the removal of β-arrestin dependent chemokine-bound CCR5 via clathrin-mediated endocytosis^28^ and relocation of receptors to recycling endosomes and/or the trans-golgi network^3^. We investigated the ability of IH-CCL5 to induce CCR5 downmodulation using a flow cytometry-based approach assessing the loss of binding for a monoclonal anti-CCR5 antibody (MC5), which recognises an extracellular *N*-terminal CCR5 linear epitope not affected by chemokine binding and CCR5 conformational changes^25^. This assay indirectly monitors the removal of cell-surface CCR5 receptors upon agonist stimulation^35,41^ and was used to compare the effect of IH-CCL5 and commercial CCL5. Initial titration of commercial and IH-CCL5 (1-100 nM) indicated comparable dose-dependent effects on MC5 binding after 15 min of treatment (supplementary Fig. 6). Both commercial and IH-CCL5 used at 100 nM showed a significant reduction in MC5 binding after 1 h of treatment, which was abrogated when cells were pre-treated with the CCR5 antagonist TAK-779 (Fig. 5a-c). In these experiments, IH-CCL5 was as effective as commercial CCL5 with 77.91% +/- 5.00% versus 74.49% +/- 2.99% of downmodulation induced by 60 min, respectively (Fig. 5d). Immunofluorescence microscopy (IF) was also utilised to investigate CCR5 internalisation upon chemokine stimulation (Fig. 5e). CHO-CCR5 cells pre-labelled live with MC5 were incubated at 37^o^C in the presence or absence of 100 nM commercial or IH-CCL5. After fixation and permeabilisation, MC5-labelled CCR5 was detected with a fluorescent secondary antibody. Figure 5e demonstrates that treatment with either commercial or IH-CCL5 leads to CCR5 internalisation, as indicated by the loss of peripheral cell-surface staining and appearance of the perinuclear staining pattern characteristic of recycling endosomes^3,35^. Such relocation was abrogated when cells were pre-treated with TAK-779, as shown for IH-CCL5 (Fig. 5e), and thus further confirms the ability of IH-CCL5 to promote the CCR5 desensitisation process.

## Discussion

In this study, we have demonstrated the successful production of CCL5 that precludes the requirement for inclusion body purification and yields high levels of recombinant CCL5. We confirmed the biological activity of the IH-CCL5 through comparing different stages of chemokine-mediated CCR5 activation, using knowledge that has been predefined in immune and transfected cell models^26–28,35,41^.

Purification of CCL5 is challenging due to the presence of structural disulfides that are difficult to form with control, leading to protein solubility issues. Although early purification of CCL5 from inclusion bodies with good yield has been reported^24^, many studies since have suffered from low purification yields due to solubilisation of inclusion bodies with high concentration of guanidine hydrochloride, causing complete denaturation of protein secondary structure, resulting in aggregate formation during refolding and thus loss of protein^42^. This includes the purification of CCL5 in a yield of 0.2 mg/L^14^ and purification of His-CCL5 where increased proportion of the CCL5 was present in the precipitate and low levels of CCL5 were soluble (no yields reported)^43^. Interestingly, an early approach to access the chemokine utilised solid peptide synthesis to construct CCL5 (1–68), and resulted in a yield of 460 mg of crude CCL5 from small proportion of an initial 0.25 mmol reaction scale^44^. But akin to expression into BL21 *E. coli*, the isolated peptide required refolding affording a final yield of 9.7 mg of CCL5, which was determined to be active by virtue of inducing the release of calcium in monocytes^44^.

Recently solubility tags were also trialled with CCL5, including construction of an MBP fusion protein^16^, which resulted in the recovery of 66.8 mg MBP-CCL5 from a 2 L *E. coli* culture. However, this large tag was not cleaved and therefore could interfere with CCL5 binding and therefore downstream CCR5 activation events. Importantly, the cleavage of solubility tags can result in low CCL5 recovery, with a reported yield of 2.5 mg/L after cleavage of MBP, lanthanoid binding and strep tags^14^.

However, a high yield of production is only useful if associated with full functionality of recombinant CCL5. Usually, the bioactivity of a produced recombinant chemokine is evaluated based on one main assay, with examples for CCL5 of either binding^16^, intracellular calcium release^44^, cell migration^14^ or long term cellular effects such as transcriptional changes and proliferation^43^. Here, using a series of assays measuring CCL5-mediated activation of CCR5 we demonstrated full functionality of IH-CCL5 in terms of receptor binding, phosphorylation, calcium flux and chemotaxis, showing that its activity was comparable to commercially available CCL5. Note that purified IH-CCL5 resuspended at 100 µM in PBS retained its functional activity after 12 months of storage at -80 °C. Additionally, since our purification method leads to a low level of endotoxin contamination similar to commercial-grade CCL5 (see material and methods), IH-CCL5 should be tolerated for long term live cells treatment studies minimising off-target effects.

In conclusion, this study details an optimised, high yielding method for CCL5 production that avoids the purification of CCL5 from inclusion bodies. Expression of CCL5 using a His-SUMO solubility tag in SHuffle *E. coli* vastly increased the expression of soluble protein and the His-SUMO tag removal did not affect the purification yields. Most importantly, the purified IH-CCL5 is biologically active and as able as its commercial counterpart to trigger the anticipated mechanistic response of CCR5 whether it is over-expressed in transfected cells or endogenously present on immune cells^3,26,35,41^. Overall, our adapted method enables high yielding, simpler, less resource and labour-intensive production of recombinant CCL5, a valuable tool to investigate the role and activation mechanisms of CCL5 receptors (CCR1, 3 and 5).

## Materials and methods

### Reagents and antibodies

All materials were purchase from Thermo Fisher Scientific, unless otherwise stated. Secondary antibodies AlexaFluor-647 (A647) conjugated GAM IgG and AlexaFluor-568 (A568) conjugated RAM IgG were purchased from Invitrogen. Mouse monoclonal antibodies (mAbs) anti-CCR5 antibodies used within this study were 2D7 (IgG2a) obtained from BD Pharmingen, and MC5 (IgG2a) purified from hybridoma as previously described^41^. Anti-CCR5 E11/19-APC (IgG1) specific for CCR5 phosphor Ser349 used for phosphor-Flow analysis was purchased from BioLegend. Histone H3 mouse monoclonal antibody (clone 1A2A3) was purchased from Proteintech. Commercial recombinant human RANTES (CCL5) produced in E. Coli (with endotoxin level guaranteed ≤ 1 EU/ug) was purchased from Peprotech. The calcium indicator (Fluo-8 AM) was purchased from Stratech Scientific.

### Cell culture

CHO cells (DHFR-deficient) stably expressing human CCR5 (CHO-CCR5 cells, gift from Prof. Matthias Mack, University of Regensburg, Germany)^26^ were cultured in Minimum Essential Medium α supplemented with 10% FBS, 10,000 units/ml penicillin, 10,000 µg/ml streptomycin and 2 mM L-glutamine). Cells seeded in 10 cm tissue culture-treated dishes were detached using Trypsin/EDTA or 10 mM EDTA-PBS, for passage or experiments, respectively.

### CCL5 expression and purification

The Human CCL5 sequence containing plasmid (see supplementary Fig. 1) was transformed into *E. coli* SHuffle T7 Express lysY and placed under ampicillin selection. Positive clones were inoculated into 1 L 2xYT growth media (with 100 µg/mL ampicillin) and induced at and OD_600_ of 0.7 with 0.2 mM isopropyl β-d-1-thiogalactopyranoside (IPTG). The culture was grown for 4 h at 30 °C post induction. Bacterial pellets were resuspended in Buffer A (80 mM Tris pH 8.2, 0.5 M NaCl, 30 mM imidazole) supplemented with protease inhibitors and sonicated. Cell lysates were clarified by centrifugation at 38,000 x g for 45 min and loaded onto a 5 ml HisTrap FF crude column pre-equilibrated with buffer A. The column was washed with 10 column volumes of buffer A followed by a gradient over 6 column volumes to 100% buffer B (80 mM Tris pH 8.2, 0.5 M NaCl, 500 mM imidazole). The eluate was dialysed into 80 mM Tris pH 8.2, 0.5 M NaCl, 10% glycerol at 4 °C overnight. The dialysed His-SUMO-CCL5 protein was incubated with SUMO protease ULP1 produced and purified as previously described^45^, a 10 mg/ml stock solution stored at -80^o^C and used at a 1:25 ratio for cleavage at 4 °C with mild agitation. The cleavage reaction was completed after 24 h and was monitored using SDS-PAGE analysis after Coomassie staining. The cleaved protein was loaded onto a 5 ml HisTrap FF crude column pre-equilibrated with buffer AB (80 mM Tris pH 8.2, 0.5 M NaCl, 10 mM Imidazole). The column was washed with 6 column volumes of buffer AB followed by a step gradient to 100% buffer B for 5 column volumes. SDS-PAGE was used to determine the purity of the fractions. The protein was dialysed in sodium acetate pH 4.5 and 500 mM NaCl overnight, in 1% acetic acid overnight, in 0.1% TFA overnight and then lyophilised (stored at -80 °C). Lipopolysaccharide (LPS) Endotoxin contamination was assessed using a LAL (limulus amebocyte lysate) assay and found to be ≤ 1 EU/ug, a level that was reduced further using an endotoxin removal column. For biological activity testing, lyophilised IH-CCL5 was dissolved in PBS and prepared as a 100 µM stock solution, snap freeze, aliquoted and stored at -80 °C until usage.

### LC-MS method

High Performance Liquid Chromatography-Electrospray Ionization Mass Spectrometry (LC-MS) of protein was performed using a Dionex UltiMate^®^ 3000 Ci Rapid Separation LC system equipped with an UltiMate^®^ 3000 photodiode array detector probing at 250–400 nm, coupled to a HCT ultra ETD II (Bruker Daltonics) ion trap spectrometer, using Chromeleon^®^ 6.80 SR12 software (ThermoScientific), esquireControl version 6.2, Build 62.24 software (Bruker Daltonics), and Bruker compass HyStar 3.2-SR2, HyStar version 3.2, Build 44 software (Bruker Daltonics) at CoEMS. Mass spectrometry was conducted in positive ion mode. Protein samples were prepared in 1:1 water: acetonitrile + 1% formic acid (v/v/v). Protein samples were analysed without the use of a column at a flow rate of 0.25 mL min − 1 at RT with a mobile phase composing of water with 0.1% (v/v) formic acid (solvent A) and acetonitrile with 0.1% (v/v) formic acid (solvent B).

### Gel electrophoresis

Samples for the protein purification gels were created with 5x reducing sample buffer, boiled at 95^o^C 10 min and resolved on a 12% or 15% SDS-PAGE gel. Gels were fixed (50% ethanol and 10% glacial acetic acid) 1 h, then stained in Coomassie (0.1% Coomassie brilliant blue R-250, 50% ethanol and 10% glacial acetic acid) 0.5 h and finally destained (40% ethanol and 10% glacial acetic acid). Samples for western blotting were created with reducing sample buffer but were non-boiled. Samples were resolved on a 10% SDS-PAGE gel.

### Chemokine binding competition assay

CHO-CCR5 cells were plated at a density of 75,000 cells/well into a 96-well plate in Binding Medium (BM: RPMI 1640 without carbonate or glutamine, 0.2% (W/V) BSA, 10 mM HEPES adjusted to pH 7), before being incubated for 2 h with or without 100 nM IH-CCL5 at room temperature (18^o^C), before being fixed in 1% formaldehyde O/N at 4^o^C. Cells were then washed and labelled in FACs Buffer using the anti-CCR5 mAb 2D7 at 5 µg/ml for 1 h, before being stained with fluorescent secondary antibody (goat anti-mouse 647). Chemokine binding is indirectly detected by loss of cell-associated 2D7 specific fluorescent signal using a CytoFLEX S.

### Western blotting

Proteins were separated on a 10% SDS-PAGE gel and transferred onto a nitrocellulose membrane. Total protein was assessed using PonceauS. Nitrocellulose membranes were then blocked in 5% milk in 0.1% PBS-Tween20 for 1 h at RT and then primary antibody incubation overnight at 4 °C. Membranes were washed 3x in PBS-T and incubated in HRP-conjugated secondary antibody (1:5000) for 1 h at RT and detected using ECL Western reagents on iBright CL1500 software version 1.8.0. Primary anti-CCR5 MC5 used at 1 µg/mL and histone H3.

### CCR5 phospho-flow

CHO-CCR5 were detached using 10 mM EDTA-PBS and resuspend at 2 × 10^6^ cells/mL in BM supplemented with phosphatase inhibitor. Cells were aliquoted equally into three polypropylene tubes (untreated, IH-CCL5 and IH-CCL5/Maraviroc) and were treated accordingly. Maraviroc (1 µM) was added prior to chemokine and allowed to bind at 4 °C for 1 h. Likewise, cells were incubated at 4 °C post 100 nM CCL5 addition for 0.5 h. Cells were stimulated at 37 °C and at the required timepoint, cells were removed and fixed in equal volume of 6% formaldehyde solution in PBS for 20 min. Cells were washed 1x with FACs buffer (1% FBS & 0.05% sodium azide) and kept in ice-cold methanol − 80 °C until staining. Cells removed from − 80 °C and washed 3x with FACs buffer and incubated in E11/19-APC (as recommended by the manufacturer) for 1 h 4 °C. Cells then washed 3x in FACs buffer and resuspended in 200 µL FACs buffer and analysed using a CytoFLEX S flow cytometer (Beckman Coulter).

### CCR5 calcium flux

CHO-CCR5 cells were loaded with 2.5 µM Fluo-8 AM and resuspended in Hanks Balanced Salt Solution (HBSS) containing 1.26 mM calcium prior to experiments. Cells intended to be treated with both CCL5 and CCR5 antagonist (TAK-779) were pretreated with 800 nM TAK-779 1 h at 4 °C. Samples were analysed on a CyAn flow cytometer (Beckman Coulter) with an output wavelength of 488 nm. Cells were aspirated for 40–60 s and baseline fluorescence from the Fluo-8 AM was recorded. After which, the acquisition was paused, 100 nM of IH-CCL5 or commercial CCL5 was added, and the acquisition was resumed to record changes in FITC signal.

### CHO-CCR5 migration assay

CHO-CCR5 cells were detached using 10 mM EDTA-PBS and resuspended in fresh complete medium. Experimental conditions included medium only, 10 nM IH-CCL5 (lower compartment), pre-treatment of cells with 800 nM TAK-779 (upper compartment) with 10 nM IH-CCL5 (lower compartment) and 10 nM IH-CCL5 in upper and lower compartments. Cells requiring treatment with TAK-779 were pre-treated with the antagonist for 1 h at RT. 1 × 10^6^ cells in 500 µL were added to 12 mm diameter Transwell insert of 12.0 μm pore size (Corning Costar) and placed into the 12 well culture plate containing 1.5 mL complete medium. The plates were placed into a 37^o^C incubator and chemotaxis was allowed to proceed for 4 h. After which, the inserts were removed and cells migrated into the bottom chamber well were counted using a haemocytometer.

### CCR5 downmodulation assay

Downmodulation assays were performed as described previously with some adaptations^41^. In brief, Respective cells were pre-treated with CCR5 antagonist TAK-779 (800 nM) 1 h 4^o^C in BM and then treated with either IH- or commercial CCL5 (100 nM) for 1 h 37^o^C. Cells were also left in binding medium only or treated with CCL5 only (100 nM) for 1 h 37^o^C. Note – Cells were kept at 4^o^C after addition of chemokine for 0.5 h before stimulations at 37^o^C. Cells were placed on ice and were incubated with 5 µg/mL of anti-CCR5 MC5 for 1 h 4^o^C and washed before being stained for 1 h with goat anti-mouse 647. Cells were fixed in 1% formaldehyde O/N at 4^o^C, washed and cell-associated fluorescence was measured using a CytoFLEX S.

### CCR5 immunofluorescence microscopy

Immunofluorescence staining was performed with small adaptations as previously described^27^. CHO-CCR5 cells were seeded onto coverslips at least 48 h before experiment. Cells were prelabelled in BM with 5 µg/mL anti-CCR5 MC5 for 1 h at RT. CCL5/TAK-779 treated coverslips were pretreated with TAK-779 (800 nM) for 1 h at RT before adding CCL5 at 100 nM in BM. Other coverslips were treated with 100 nM CCL5 alone or left in BM. All Coverslips were placed at 37^o^C for 0.5 h, fixed in PBS with 3% formaldehyde for 0.5 h at RT and quenched in NH_4_Cl (50 mM) O/N 4^o^C. Samples were permeabilised with saponin (0.05%) at RT and Fc receptors were blocked in PBS containing 0.05% saponin and 1% FBS (0.5 h). Cells were stained with anti-mouse Alexa fluor 568 (A568, 4 µg/mL) 1 h at RT. After washes in PBS/saponin and PBS, coverslips were mounted using mowiol containing DAPI. Samples were examined with Zeiss LSM980 confocal microscope (63x/1.4 oil), single confocal sections were acquired, and images analysed using ImageJ.

### Data and statistical analyses

Data expressed as mean ± SD of the stated number of experiments and were analysed with GraphPad Prism v9.4.1. The indicated statistical tests were used, where appropriate.

## Electronic supplementary material

Below is the link to the electronic supplementary material.

Supplementary Material 1

## Data Availability

All data generated and analysed during this study are included in this published article [and its supplementary information file].
