# Supplementary material for: New insight into a simple high-yielding method for the production of fully folded and functional recombinant human CCL5
Source: Sci Rep. 2024 Oct 15;14:24188. doi: 10.1038/s41598-024-75327-y (PMC11480376; doi:10.1038/s41598-024-75327-y)
Supplement: Supplementary file 1 — Supplementary Material 1 [file 41598_2024_75327_MOESM1_ESM.pdf]

Supplementary Information

New insight into a high yielding and efficient method for the production of recombinant human CCL5

Afzaal Tufail[1,2], Saeed Akkad[1], Amanda R. Noble[1], Martin A. Fascione[1]* and Nathalie Signoret[2]*

[1]Department of Chemistry, University of York, Heslington, York, YO10 5DD, UK. [2]Hull York Medical School, University of York, YO10 5DD

Presenting figures for: Construct design; Mass spectrometry analysis of purified IH-CCL5; Full-length blot for CCR5 western blot analysis; Histograms overlays of phospho-FLOW data; IH-CCL5 mediated calcium flux analysis using a plate reader-based method; Titration of commercial and IH-CCL5 for induced loss of CCR5 surface expression.

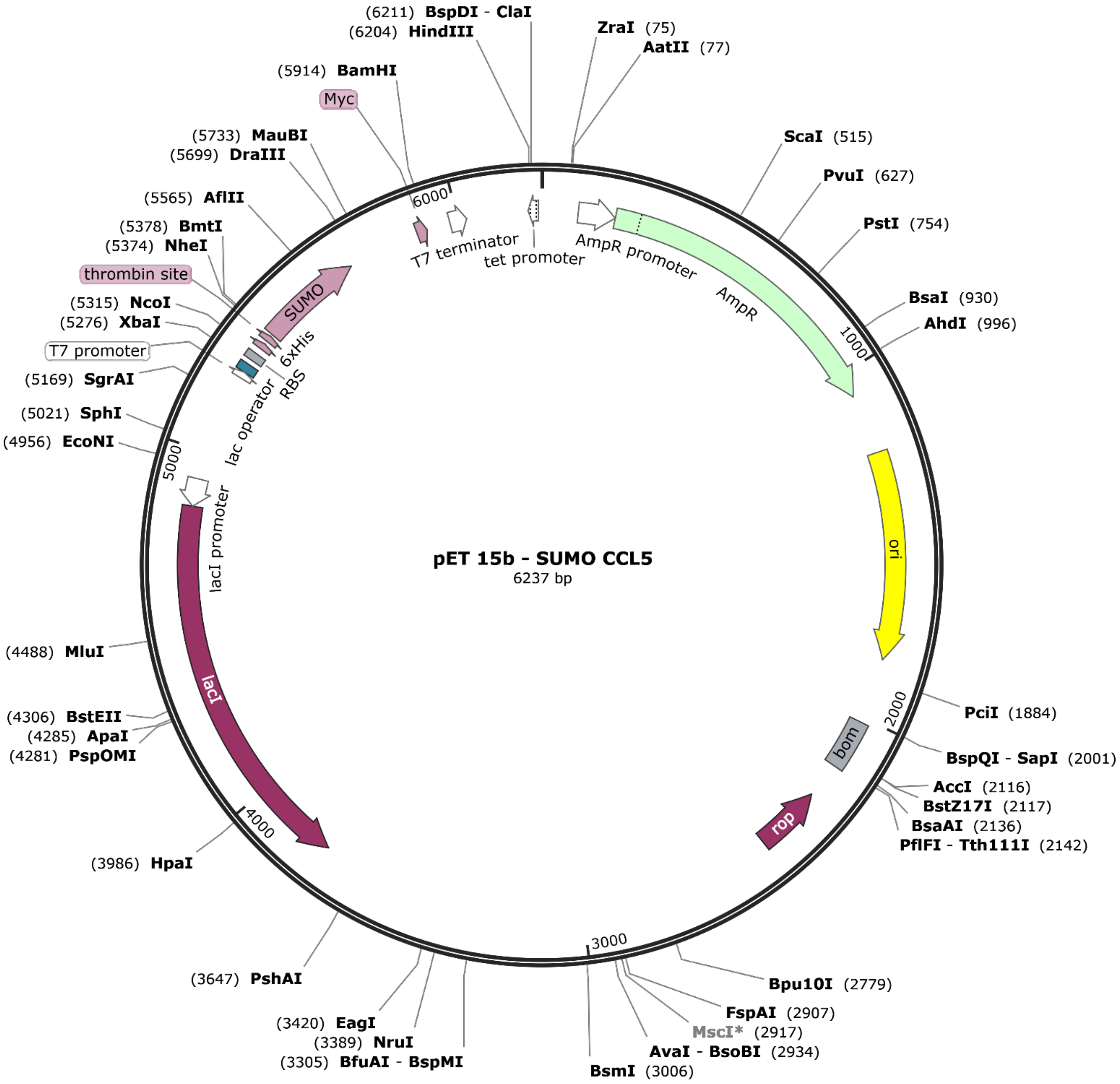

His$_6$-SUMO-CCL5 protein sequence
MGSSHHHHHHGSGLVPRGSASMSDSEVNQEAKPEVKPEVKPETHINLKVSDGSSEIFFKIKKTTPLRRLMEAFAKR QGKEMDSLRFLYDGIRIQADQTPEDLDMEDNDIIEAHREQIGGSPYSSDTTPCCFAYIARPLPRAHIKEYFYTSGKCS NPAVVFVTRKNRQVCANPEKKWVREYINSLEMS*

Supplementary figure 1. SUMO-CCL5 plasmid map

His$_6$-SUMO-CCL5 gene was purchased codon optimised from GenScript Biotech, subcloned into pET15b vector with flanked NcoI and BamHI restriction sites at the N and T termini. The Human CCL5 inserted sequence corresponds to the Uniprot sequence ID P13501 without the signal peptide (Schall, T. J. et al. 1988

J. Immunol. **141**, 1018–1025). The plasmid map shown was generated using SnapGene. The His$_6$-SUMO-CCL5 protein sequence highlight the fusion tag in blue and full length CCL5 in black. SUMO cleavage is traceless and leaves a native N-terminus serine.

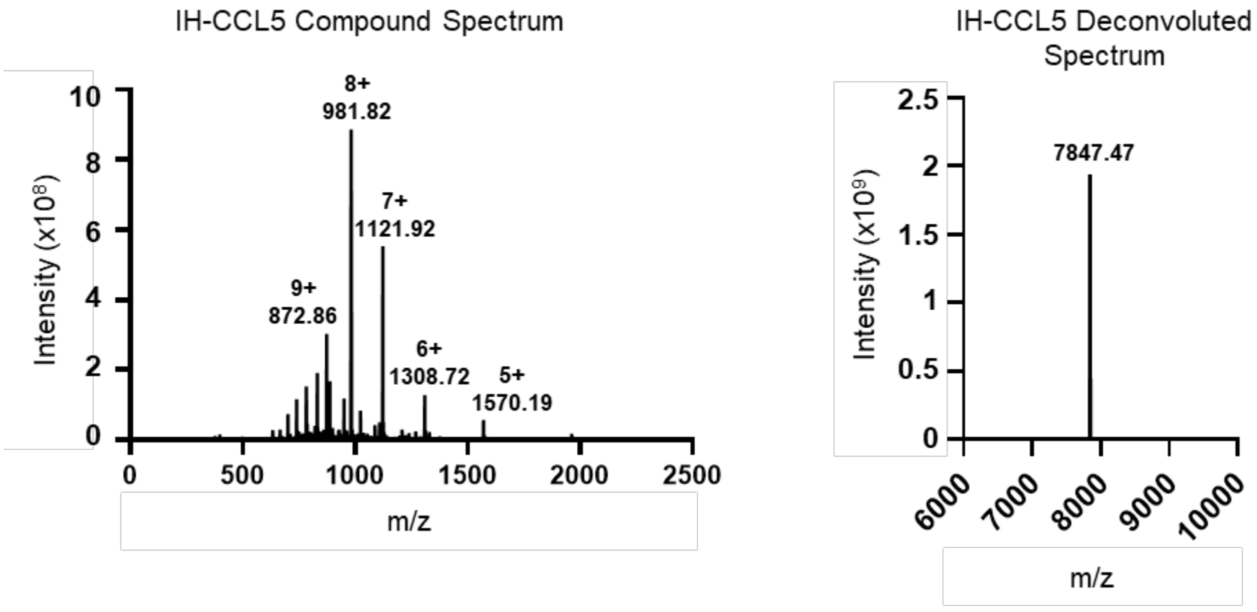

Supplementary figure 2. Positive ESI mass spectrum of purified CCL5

Protein samples were prepared in 1 : 1 water : acetonitrile + 1% formic acid (v/v/v) and run on positive ESI mode. Charge ladder for IH-CCL5 showing a mass of 7847.47 Da (required mass 7851.01 Da) indicating the formation of two disulphide bonds.

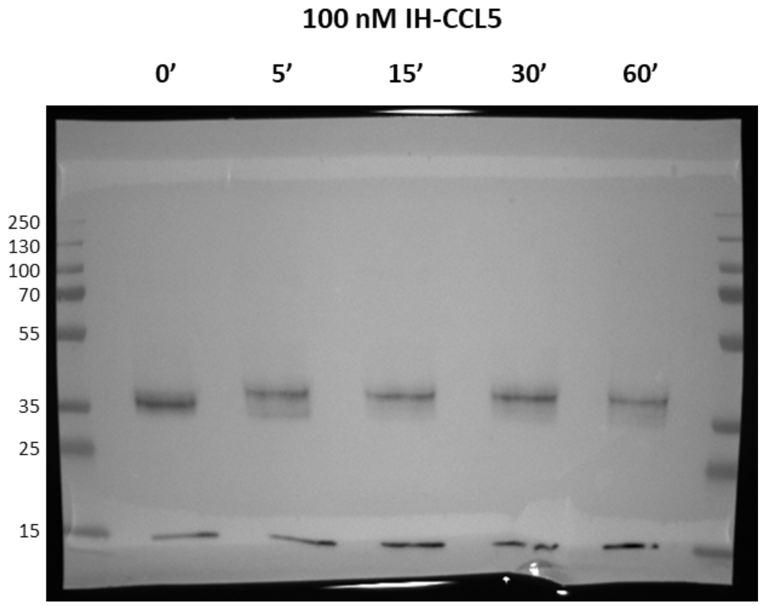

Supplementary figure 3b: Full-length blot from Figure 3 detecting CCR5 band shift after IH-CCL5 stimulation and Histone 3 loading control.

The membrane was initially blotted to detect CCR5 (37 kDa) and the band shift due to IH-CCL5 stimulation before being restained for histone H3 (15 kDa) as a loading control. Both proteins were detected with an anti-mouse HRP antibody.

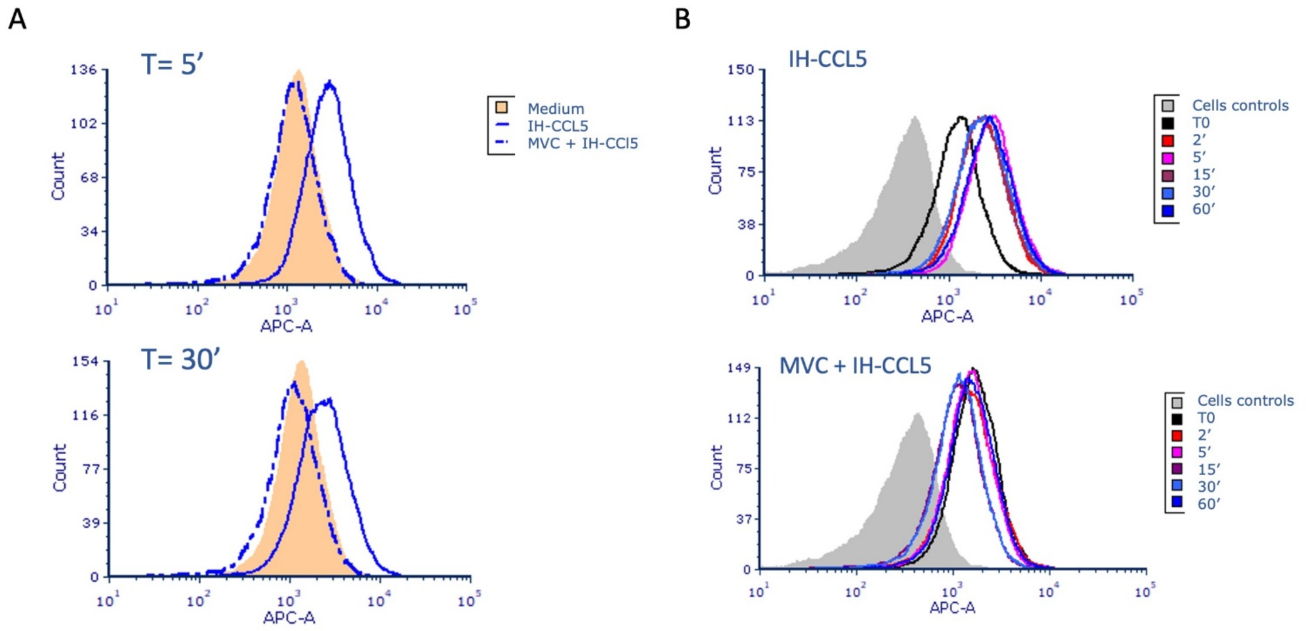

Supplementary Figure 4. Flow cytometry histograms overlays of phospho-FLOW data used to calculate the fold changes in E11/19-APC signal for Figure 3C: A. The shift in E11/19-APC signal induced by IH-CCL5 5' or 30' stimulation is not seen when cells are pre-treated with the antagonist Maraviroc (MVC); B. MVC pre-incubation prevented the shift in E11/19 signal seen with IH-CCL5 alone throughout the duration of treatment.

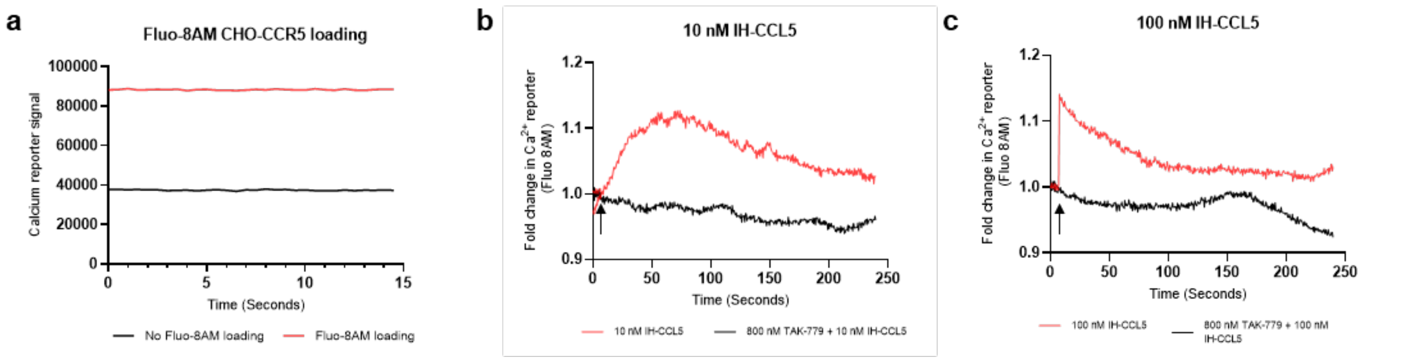

Supplementary figure 5. Plate reader based calcium assay using IH-CCL5

CHO-CCR5 cells were seeded at 10 x10^3 cells into a 96 well plate and allowed 48 h to adhere. Cells were loaded with 2.5 µM Fluo-8 AM (Stratech) and then 100 µL of Hanks Balanced Salt Solution (HBSS) containing 1.26 mM calcium was added. Cells requiring TAK-779 treatment were pretreated with 800 nM TAK-779 for 1 h. CLARIOstar Plus (BMG Labtech) plate reader was used with filter setting of 490/525 nm with cut-off 515 nm. Cells were stimulated with IH-CCL5 and a kinetic of calcium flux was recorded over time. Arrows on graph shows time at which IH-CCL5 was added.

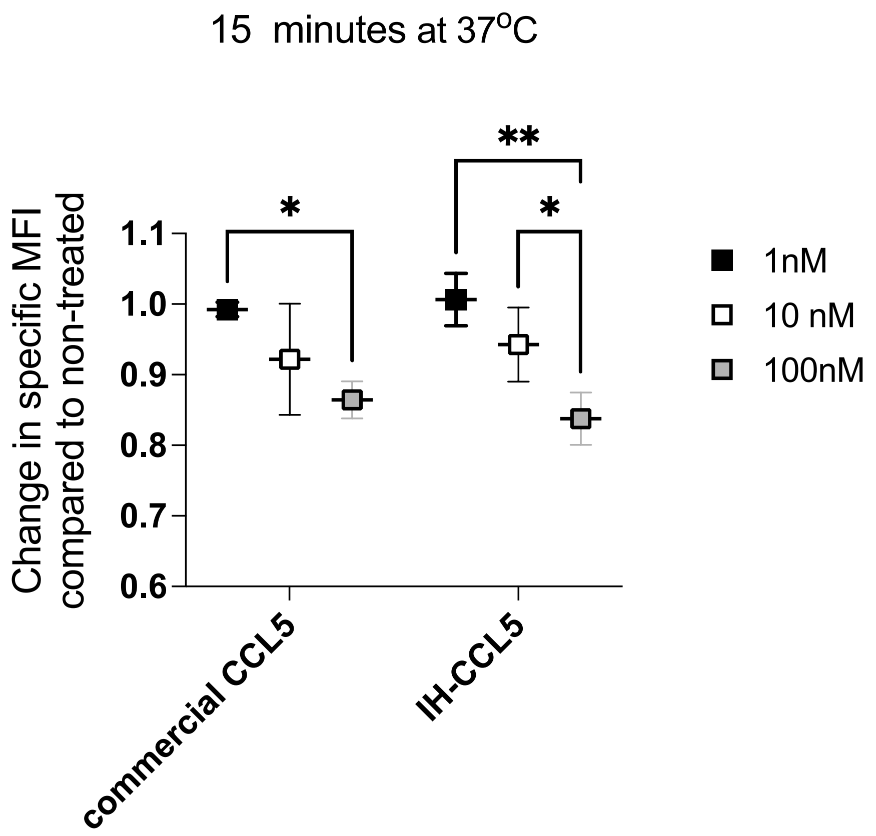

Supplementary figure 6: Titration of commercial and IH-CCL5 for induced loss of CCR5 surface expression.

CHO-CCR5 cells were for downmodulation assay as described in the material and method section of the main manuscript but threated with increasing concentrations (1-100 nM) of commercial or IH-CCL5 for 15 minutes to measure early chemokines effects. This is a representative experiment carried out in triplicate. ** $P \leq 0.01$, * $P \leq 0.05$ two-way ANOVA with secondary Bonferroni's multiple comparisons test.
